# Challenges of 20th century Ethiopian Science education

**DOI:** 10.1016/j.heliyon.2021.e07157

**Published:** 2021-05-28

**Authors:** Desta Berhe Sbhatu

**Affiliations:** Mekelle Institute of Technology, Mekelle University, PO Box 1632, Mekelle, Ethiopia

**Keywords:** Ethiopia, Science education, Secular education, Relevant and quality education

## Abstract

State-led secular education was introduced to Ethiopia by the second half of 1900s. Some studies have looked into the development the secular education system. But the historical development of science education system was not explored. This review looked into the initiatives, achievements, and challenges in introducing and advancing science education in the 20^th^ century. For this purpose, research reports, books, official government documents, and other relevant literature, and instructional materials released before mid 2000 were explored. Accounts of several researchers and official documents demonstrated that the various initiatives by the four subsequent regimes of Ethiopia to build relevant and quality science education system did not come up with expected results. By the dawn of the 21^st^ century, the country was struggling to build relevant and quality science education system. Hence, this article is prepared and presented to demonstrate the critical historical challenges in putting relevant and quality science education system in place and assist policy-makers and practitioners in formulating better policy directions and developing workable science education programs and projects.

## 1 Introduction

A succinct Tigraian wise saying that goes “ዘይተምሃረ፥ ዘይተምሓረ!” which can be loosely translated as “*one who is not educated is not liberated*” explains a lot about the use of education to humankind. As often rightly regarded, it is one of the principal tools assisting in the overall transformation of communities, nations, and the world at large. Mindful of this verity, ancient kingdoms and modern governments of the world endeavored to introduce and develop education to drive and support the politico-cultural, socio-economic, and scientific-technological lives of their societies. Some medieval Ethiopian communities and institutions were known to have adapted some sort education system. However, the history of Ethiopia's government-mandated secular education starts in the first decade of the 20^th^ century. Historical accounts of Ethiopia's secular education show that several initiatives have been taken to build relevant and quality education system, in general, and relevant and quality science education system, in particular, throughout the 20^th^ century. However, the quest for building relevant and quality national education system − a system that reflects and responds to individual and social needs, expectations, and aspirations of the Ethiopian people − was, in part, an elusive enterprise.

Twenty years into the 21 century, in spite of the recent commendable successes in expanding access to education across regions and in all levels, efforts aiming at improving the relevance and quality of the education system are not yet coming with encouraging results. The most resent status report developed to present strategic directions for the preparation of the “Ethiopian Education Development Roadmap (2018–2030)”, has discussed at length about the challenges facing the country in building relevant and quality education systems across levels, disciplines, and trades including science [1]. Since the challenges of developing relevant and quality science education are persisting, bringing its history and challenges into light helps in guiding future reforms and designing initiatives accordingly. Thus, the goal of this article is to summarize and present the history of 20^th^ century Ethiopian science education with the intention of assisting policy-makers and practitioners in the subject.

## 2 Data collection and organization

Data and information utilized in developing this manuscript were acquired through document study. The documents were collected from libraries, friends, and websites as hard and electronic copies. They include books, academic research articles, national education status review reports, national policy and strategy documents, education development roadmaps and program action plans, national reform directives and proclamations, discussion papers, reports of African education conferences, and upper primary (grade 5 to 8) science textbooks of Tigrai State, Ethiopia. The documents, except the upper primary science textbooks, were studied to extract data and information about the historical accounts of the 20^th^ century Ethiopian education, in general, and science education, in particular − with special focus on the achievements and challenges in building relevant and quality systems. The upper primary science textbooks of Tigrai − which were essentially identical to the science textbooks of all regional states of the country except the languages in which they are written − are used to conduct a brief case study on the nature of the science inquiry activities designed for that level. They were also convenient for the author because he lives in Tigrai; has excellent command of Tigrinya language, and was involved in projects and programs of advancing science education in the state. Some impressions and observations of the author, as a student and secondary school biology teacher in the system, are also included to corroborate the documented challenges and observations of researchers on the 20^th^ century Ethiopian science education.

The content is, then, organized and presented in three sections. The first section presents a very brief account of Ethiopia's pre-secular education systems with some emphasis to the education system of the Ethiopian Orthodox church. The second section presents a concise account of Ethiopian secular education system by presenting the major efforts, achievements, and challenges it faced during the four regimes that governed the country. The last section details how science education is incorporated, the efforts aiming at introducing and developing relevant and quality science education, and the principal achievements and challenges in that regard. The article is closed by a very brief concluding remarks and recommendations. Generally, this piece of work is presented by highlighting the key achievements and critical challenges to assist in formulating better policy directions and selecting and designing workable science education programs and projects.

## 3 Presentation of the findings

### 3.1 Pre-secular Ethiopian Education

The roles of various community, Koranic and pre- and post-1900 foreign missionary schools in providing education opportunities to children are worth recognition. The two Lazarist Catholic Missionary schools, namely Tsinseta-Mariam (established in 1844 in Gol'a, Adigrat) and Lideta-Mariam (established in 1845 in Alitena, Irob), are the oldest modern schools that deliver religious and secular education [2]. But the Ethiopian Orthodox church receives a special place for its role in the foundation of Ethiopia's secular education without denying the contributions of these religious and community schools [3]. Thus, owing to its influence for the beginning and development of secular education, it is fair and helpful to provide a brief background of Ethiopian Orthodox church education system.

The foundation of Ethiopian church education is linked to the introduction of Christianity during the Axumite period (4^th^ century AD) [3, 4, 5]. The content of the ‘curriculum’ of Ethiopian Orthodox church education system includes Ge'ez and literature, poetry, church music, world history, mathematics, philosophy, Biblical exegesis and history, doctrine, history of the church, liturgics, civil and canon law, Christian ethics and pastoral theology [3]. Even though this educational system lacks written curricular guidelines, it is structured into various distinctive hierarchical levels, each of which requiring students to demonstrate specific sets of expertise which can take up to 30 years or more to complete [5, 6]. The various levels of Ethiopian Orthodox church education system include School of Reading, School of Holy Mass, School of Hymns, School of Poetry, and School of Scriptures [5]. While the School of Reading and School of Holy Mass are regarded as basic and accessible to many people, the School of Hymns, the School of Poetry, and the School of Scriptures are equivalent to tertiary level education of any modern educational system where very few determined, talented and gifted people would pursue them. At the higher level, the content of the curriculum leads to specialization.

In the primary level of education, reading and recitation of religious texts are the dominant methods of instruction [6]. In school of Holy Mass, students learn through voice, hearing and movement. School of Poetry requires students to be engaged in a lot of minds-on activities to understand and develop ambiguous, vague and secretive expressions [3]. One important component of teaching and learning, i.e., writing, is limited; hence, not many people who can read would be able to write with ease. This may be because writing was restricted to religious matters but not for public purposes [6]. Females are not involved in church education, though the system is theoretically open for all children of Christian faith for free [5]. It was only during the 19^th^ century that very few girls were reported to have been getting to the schools to learn reading [6].

The principal objective of the Ethiopian Orthodox church is to deliver religious education, produce priesthood, and preserve and diffuse Christian culture. The system strives to produce people who accept and live with the existing, culturally and historically acceptable norms and traditions and pass that knowledge without altering it, but not who strive to understand nature [3]. Besides to providing religious education, church schools are also responsible for promoting cultural, spiritual, literary and scientific knowledge. Monasteries and churches of Ethiopian Orthodox were the centers of instruction and research. They were centers of artistic works, ethnomedicinal studies, and preparation and production of manuscripts of religious, cultural, administrative, and historical significance. Moreover, Ethiopian Orthodox church education was responsible for producing civil servants such as judges, governors, scribers, treasurers and public administrators. Most of the educated personnel who were serving in government, public and religious sectors of pre-World War II Ethiopia were the products of this system of education [3, 5]. However, at the beginning of 1900, the need for modern education became apparent, which led to the introduction of secular education in mid-1900.

### 3.2 Brief historical account of Ethiopian secular education

The 100 years history of the Ethiopian state-mandated secular education shows us that it had always been ailing. Though the four regimes of the 20^th^ century Ethiopia had come up with their own peculiar initiatives to develop the secular education system, the earlier three regimes have left the scene by leaving the challenges they inherited from their predecessors and those created by their own. Thus, it is proper to present the historical account of the secular education in four parts corresponding to the four regimes.

#### 3.2.1 The inception – Education in the reign of Menilik II

Ethiopia's long history of isolation and the excessively conservative culture of the Orthodox Church were argued to be the reasons for lack of interest in secular education earlier than the beginning of the 20^th^ century [7]. In the middle of the 19^th^ century, few Ethiopians had the opportunity of foreign scholarship through European missioners. Two Lazarist Catholic Missionary schools were also operational in eastern Tigrai, north Ethiopia [2]. However, the influence of foreign-educated Ethiopians was not significant to initiate secular education. In fact, foreign educated Ethiopians of the mid- and late-19^th^ century were seen suspiciously for their affiliation to foreign culture and religion. But towards the beginning of the 20^th^ century, the need for modern secular education was no more to be ignored. By the mid 1900s, Emperor Menilik II learned that secular education is essential for his government. In 1905, he had already started a school in his palace for children of the nobility [3]. By the same year, he issued a proclamation that urged parents (i.e., the nobilities in practical sense) to send their children to schools while promising that his government prepares the schools and teachers [3, 7]. The Emperor, in collaboration with Abune Mathewos (the then head of Ethiopian Orthodox Church) hired teachers from Egypt and opened the first four schools in 1906 [3,5,[7], [8], [9]]. The curricula included Amharic, Ge'ez, French, English, Arabic, Italian, mathematics, science, and physical training and sports [3].

The beginning and survival of secular education during its early stage can be regarded as laudable historical achievement. The best way to evaluate that achievement is to look into it through the spectacle of the challenges of that time. Many scholars who provided the historical accounts of secular education in Ethiopia mentioned one or more of the following factors that impeded its development [3, 4, 5, 7]. The challenges can be summarized as follows. (1) Most Ethiopians of that time had negative attitudes towards skilled manual labor, hence Western education. Neither the masses nor the nobility had any knowledge about the importance of any education outside that of the Orthodox Church. (2) The schools were staffed by teachers from the Egyptian Copts for fear that teachers from elsewhere might interfere in religious affairs. (3) The people considered that sending one's children to the schools to be against their culture and values. Since the teachers were mostly foreigners, the people in the orthodox-dominated part of the country regarded the schools as centers of Catholicism. (4) The illness (1906–1908) and the subsequent death (1913) of the Emperor left the infant secular education unattended. The death of the Emperor left the country in the hands of squabbling feudal lords where no significant achievements were apparent in all sectors until 1928.

But these accounts ignore or fail to acknowledge two important matters. First, Menilik's Ethiopia incorporated many previously independent nations in the east, west and south through horrendous military campaign. Hence, the people of the nations in the newly incorporated parts of the country cannot be regarded as supporters of Menilik and his initiatives. Second, the schools of Menilik II could not be considered as accessible to children of all citizens. The schools were neither intended nor accessible to the children of the oppressed and dispossessed communities. Therefore, when Ras Tafari Mekonnen (later Emperor Haile Selassie I) took power in 1928, there was only a single modern school in the country. And yet, despite the efforts of Emperor Haileselassie I to promote the development of education, the Italian war of aggression destroyed all the achievements in terms of material and manpower. Hence, when the Italian war of aggression was averted in 1941, the restoration of secular education was begun anew.

#### 3.2.2 The foundation – Education in the reign of Haileselassie I

The real rehabilitation of education was started by the beginning of 1942. The major efforts to restore and then develop the education system between 1942 and 1973 were listed by Kiros [4] as shown in Table 1. It is apparent that appreciable achievements were registered during the decade of 1940s. The foundation of Ethiopia's modern educational system was laid during that decade [3]. Nonetheless, there were a number of inherent problems. Readers need to note that the attempt to indicate the problems is critical. But in the opinion of the present author, the unfortunate historical reality about the development of the Ethiopian secular education is the failure of the Ethiopian Orthodox church education system to evolve into modern secular one. The contribution of Orthodox Church education in the foundation and development secular education was marginal. This left the empire with no other choice than adapting educational systems of Western societies. And yet, it was very apparent that any imported educational system had a number of problems.

**Table 1:** Major achievements in the reign of Emperor Haile Selassie I [4].

Years	Major Achievements
**1941-1950**	
1941	Establishment of the Ministry of Education and Fine Arts
1943	Beginning of secondary education (Haile Selassie I High School established)
1943	The power of the Minister of Education and Fine Arts defined (along that of other ministers)
1944	Establishment of teacher training schools, Commercial and Technical School of Addis Ababa
1947	Establishment of National Board of Education
1947	Issuance of proclamations and directives to empower and financially support education
1948	Efforts to develop adult education began: creation of *Birhanih Zarie Naw* Institute
1950	The first higher education – University college of Addis Ababa started classes
**1951-1960**	
1951	Establishment of Committee on Haile Selassie I University
1953	Establishment of Long-term Planning Committee for the development of Ethiopia's education.
1952–4	Engineering, building technology, agriculture and public health colleges established
1957	First Five Year (1957–1962) Education Development Plan adopted
**1961-1973**	
1961	Issuance of the Charter Haile Selassie I University to place all colleges under the umbrella of one university
1962	UNESCO supported technical study on investment needs of the Second Five Year Development plan
1963	Issuance of Second Five Year (1963–1967) Education Development Plans
1968	Issuance of Third Five Year (1967–1973) Education Development Plans
1972	Education Sector Review: Comprehensive and critical review of the educational system

Despite these initiatives, the Ethiopian education system during this foundation phase was marred with a multitude of problems. According to the accounts of many researchers [3, 4, 5, 7], the most notable problems include the following: (*a*) Most of the teachers were foreigners – English, French, Italians, Canadians, Indians, Egyptians, and Americans. (*b*) Native teachers and other educational personnel were lacking to teach and lead the educational system. (*c*) There was high attrition of teachers due to low payment and unattractive working situations. (*d*) The contents and instructional approaches were copies of Western education system – that were concentrating on academic contents and giving little emphasis to technical fields. (*e*) Amharic and English languages were the media of instruction though Amharic was unfamiliar to the great majority of the population and English was alien to all. (*f*) Schools as well as educational facilities and supplies were meager. (*g*) Print materials in Amharic and any of the Ethiopian languages were lacking. (*h*) Educational access was characterized by regional, rural-urban and gender disparities. (*i*) The educational administration was over-centralized with inefficient bureaucracy. (*j*) Students seeking enrolment at various levels of the education system were growing beyond the capacity of the system and the state.

These problems became apparent when the Addis Ababa UNESCO (United Nations Educational, Scientific and Cultural Organization) Conference came up with shocking evaluation about the status of Ethiopia's education [10]. Nearly 20 years after the foundation of modern educational system was put, a historic UNSECO Conference at Addis Ababa (15–25 May, 1961) came up with bad news about the status of Ethiopia's education. It revealed that Ethiopia's education was still among the most backward systems in Africa. This led to the evaluation of the education system, which was realized by the launching of the Education Sector Review (ESR) in 1971 [4]. The Sector Review identified the following problems as listed in the work of Kiros [4], which were the summarized versions of the aforementioned ones. They were: alienation of the Ethiopian children by means of imported education system; disregard to the Ethiopian diversity through elitist and rigid education system; educational wastage at all levels; inequitable distribution of education opportunities, and over-centralized and inefficient of educational administration. Based on these findings, the Sector Review put the following recommendation:

Education must aid in the transformation of the Ethiopian society, by playing a vital role in the lives of all citizens. To do this, the present educational system must be restructured and changed. Education must be conceived in its broadest connotation to include all non-formal and formal learning experiences. It must take advantage of new technology, and of social and religious institutions, so that education can be delivered to the Ethiopian population as a whole (p. 85) [4].

According to Kiros [4] and Negash [11], had the proposals of the Sector Review been implemented, it could have led Ethiopia along the right track. However, when the urban population (teachers, students and parents) critically opposed the recommendations of the Sector Review perceiving that it was against their interest, no significant effort was made by the Ministry of Education and Fine Arts to explain that, in fact, the recommendations do not favor one group against another. Many people agree that the opposition served as a precursor for the overthrow of the government of Emperor Haile Selassie in 1974 [8].

#### 3.2.3 The crisis – Education in the Dergue regime

The Imperial government of Haile Selassie I was abolished in September 1974. Since the problems of the education system were among the reasons for the downfall of the Imperial regime, it would be apparent that a regime that follows would politicize it. Without acknowledging the efforts of the Imperial regime towards correcting the problems of the empire's education system, the Provisional Military Administration Council (i.e., the Dergue), developed a new one based on two arguments [11]. The arguments were: “the educational policy of the Imperial regime was elitist (favoring some regions and urban areas)” and “the curriculum did not take into account the concrete conditions in the country” (p. 107). The elitist and selective nature of Ethiopia's educational opportunities during the Imperial regime was apparent as was also stated by Wubneh and Abate [7]. Though written education policy was non-existent, the education opportunities were factually elitist and selective. Thus, the Dergue developed its own education system – interestingly based on the recommendations of the Sector Review [11].

The most commendable achievement of the Dergue was the launching of literacy campaign in 1979 in multiple languages, which reduced the illiteracy rate considerably [11, 12]. The major measures taken by the Dergue, which were characterized by decrees and proclamations, were summarized by Kiros [4] as give in Table 2. Another achievement that deserves to be acknowledged is the expansion of educational opportunities to rural and suburban communities. Even though the percentage increase in terms enrolment is challenged [11] some authors argue that the expansion of educational opportunities to many communities of the country by the Dergue to be commendable [4, 12].

**Table 2:** Major efforts in the reign of the Dergue (1974–1991) [4].

Years	Major Efforts
1974	Issuance of proclamations on Development through Cooperation, Enlightenment and Work Campaign
1975	Establishment of a reform group to evaluate the quality of education and to recommend change (i.e., *Yetmhrt Aqtatcha*)
1975	Issuance of proclamation on public ownership of private schools
1976	Issuance of proclamation on public administration and control of schools
1977	Issuance of proclamation on the administration of higher education institutions – creation of the Commission for Higher Education
1979	Launching of National Literacy Campaign
1984–85	Evaluative Research of the General Education System of Ethiopia presented
1984	Issuance of Proclamation to Strengthen the Management and Administration of Schools

The Sector Review of 1972 had planned to enroll 90% of school children to Minimum Formation Education (grades 1 to 4) by 1983–1984 [3]. Nonetheless, though the Imperial regime was collapsed about 10 years earlier, the trend was not promising to achieve that goal. Nearly a decade later (in 1980) the Ethiopian Ministry of Education (MoE) developed another plan to provide eight years of universal primary education to all school age children by 1986 [11]. This could not also be realized either. In general, the education system of the Dergue was, like its predecessor, also characterized by many problems – some were old and inherent and inherited while some were contemporary – and described to be in crisis [13]. The major problems of the education system of the Dergue were [4, 14, 15, 16]:

(*a*) Absence of written education and training policy as well as explicit education objectives and strategies.
(*b*) Increase of the population of junior and senior secondary school students beyond the capacity of the country's economy to support their education and to provide jobs.
(*c*) Failure to provide culturally, economically and socially relevant curriculum as promised.
(*d*) Low funding of the education sector that led to shortage of educational facilities and inputs.
(*e*) Teachers' dissatisfaction and attrition due to low payment and unsatisfactory working conditions.
(*f*) Regional and gender disparities in providing educational opportunities in the primary, secondary, and tertiary levels.

These problems, more or less, describe the state of the education system when the government of the Dergue was abolished in mid 1991.

#### 3.2.4 Democratization – Education in the federal Ethiopia

The post-1991 government began the preparation of its own education system as it assumed leadership of the country in mid-1991. It began to democratize the education system by issuing a new policy, called Education and Training Policy (ETP), in 1994 [15,16]. The policy addressed the following important problems of Ethiopia's education system that have been apparent for years. The ETP clearly stated and elaborated the general and specific objectives of Ethiopian education system. Moreover, it established the strategies of curriculum development, the levels and scopes of educational structure, the strategies of educational measurement and examination, the strategies of teacher education, the languages of instruction, the strategies of coordinating education, training and research and development, the mechanisms and strategies of supporting the education system, the guidelines of educational organization and managements, and the strategies of financing the sector. Building socially, economically and culturally relevant education system is a critical achievement in building democratic society. The policy stated the nature and scopes the curricula across all levels in relation to meeting the needs of the country and its peoples and providing education opportunities for all children. In this regard, the policy partly answered an age-old problem of language of instruction, where native languages were allowed to be used as languages of instruction in primary schools (grades 1 to 8).

As a commendable feature of the post-1991 education policy, the issue of language of instruction requires an additional treatment. This is because an attempt of providing culturally and socially relevant education begins with using learner's own languages as media of instruction. The attempt of using an Ethiopian language (i.e., Amharic) as medium of instruction during the 1940s was not successful. Even though Amharic was adopted as a language of instruction for primary schools later [3], it did not address the issue of language of instruction under the Ethiopian diverse linguistic reality. Cognizant of this reality, the government of the Dergue made some efforts to carry out the adult literacy program through a dozen of national languages [4]. Moreover, standardization of science and technology terms into Amharic had been conducted by the Ethiopian Science and Technology Commission in the mid-1980s. In fact, the desire of using African languages as media of instruction at all levels had been considered several times during the UNESCO sponsored African educational development conferences of 1961 (Addis Ababa) [10], 1968 (Nairobi) [17], 1976 (Lagos) [18] and 1981 (Arusha) [19]. One of the recommendations (Recommendation 4.1) of the 1976 Lagos Conference [18] stated:

Considering that African languages constitute the most appropriate instruments to express the genius of our peoples' and ‘that an education given in these languages offers invaluable pedagogical and cultural advantages’ the conference called for ‘the use of African languages as languages of instruction.

Hence, the beginning of using native languages as media of instruction was one step towards the process of culturizing education, which can be regarded as important characteristics of the education system. But it has to be noted that not all children of the country were taught through their own languages. Many nations, nationalities and peoples of the country were incapable of adopting their languages as media of instruction.

We have seen that one of the problems of Ethiopia's education system during the regime of Emperor Haile Selassie I was its over-centralization [4]. In fact, the 10 Year Program for the Controlled Expansion of Ethiopian Education prepared by the then Ministry of Education and Fine Arts in 1954 [20] strived for geographical decentralization of secondary schools. Some measures to address urban-rural and regional disparities of primary and secondary schools [12] and to establish regional educational planning centers [4] were also taken by the Dergue. In the post-1991 ETP, the issue of decentralization is pushed forward, where educational administration was completely decentralized even though the mandate of curricular development were barely given to the regional states. The ETP 3.8.2 states, ‘educational management will be decentralized to create the necessary condition to expand, enrich and improve the relevance, quality, accessibility and equity of education and training’ [11].

The MoE developed a twenty-year education sector indicative plan with a goal of achieving good quality universal primary education by year 2015 as per the prescription of the policy [21]. It is apparent that this goal was ambitious; though the registered achievements were quite encouraging. Nonetheless, problems of high student population per classroom, high rate of attrition and repetition, shortage of qualified secondary teachers, and increasing gender gap where females remain behind were persisting [21]. Moreover, very limited educational opportunities for kindergartners and pastoral communities, limitation of educational facilities and supplies, inadequacy of the qualities and quantities of instructional materials, and lack of quality teacher education schemes were still unabated problems.

### 3.3 100 Years of science education in Ethiopia

The Ethiopian secular education was founded on a package of imported system – imported content, imported practice, imported personnel, and imported language of instruction. This section discussed the history of science education in the 20^th^ century. According to Berry et al. [9], only rudimentary science was taught in the first ever schools of Emperor Menilik II. The development of the empire would have been realized through educating all citizens in areas of science and technical fields. But the secular education of the reign of Menilik II was focusing on training bureaucrats. Hence, not much can be said about science and science teaching before the 1940s.

#### 3.3.1 Science education in the reign of Emperor Haileselassie I

It was indicated previously that the foundation of Ethiopia's secular education was laid during the decade of 1940s – sometimes referred to as the decade of restoration. Therefore, the curricula of the 1940–50 were prepared to meet the immediate manpower needs for post-war reconstruction. Since the teachers and teaching materials were imported, the curricula were not aligned with the attributes of the Ethiopian children. The National Board of Education (established in 1947) formulated nationwide policy of uniform curricula for grades 1 through 6 [3]. Despite the intention of building culturally and socially compatible curricula, the contents as well as the approaches and contexts of teaching were alien. It was only for grade 1 and 2 that Amharic was recommended as a language of instruction − which also was and is alien to the linguistically diverse children of the country. Starting grade 3, students were required to learn science and other subjects in English. Moreover, since the textbooks and other curricular materials were written in European languages (usually English), the contexts upon which science was presented and taught were also alien [3]. The Ministry of Education and Fine Arts developed curricular guide in 1949 to standardize instructional practices for grades 7 and 8. Owing to the foreign-based instructional materials, the attempts to gear the curricula to the needs of Ethiopian students were unsuccessful. According to Wagaw [3], “the prescribed course in science made little mention of personal or community hygiene, food and nutrition, health or safety measures, and agriculture and conservation of natural resources. The course outlines were theoretical and dealt with such topics as solar system, magnetism, expansion of solids and liquids, and the like. Very little of this had any practical relevance for unsophisticated pupils in a rural setting” (p. 73).

The following decade (1950s) was regarded as the time for consolidation and expansion of what had been laid in the restoration period. The processes of consolidation and expansion were likely not easier than the processes of restoration and reconstruction. Retrospectively, it is apparent that more attention was given to quantitative expansion of educational opportunities at all levels with comparably little effort to ensure the quality of the education system. Hence, no fundamentally apparent measures were taken to make science content and science teaching more relevant to the children of the empire during the 1950s as well. In general, the quality of education was described as low [4].

The decade of 1960s was known as the Decade of Africa. As indicated in section 3.2.2, the UNESCO Conference of African States for the Development of Education at the beginning of this decade (Addis Ababa, May 15–25, 1961) revealed that Ethiopia's achievement in developing the education sector during the previous 20 years was unsatisfactory [3, 4]. The very apparent limitation of the government in this case was that it didn't attempt to compare Ethiopia's education system against other neighboring nations. Hence, the Conference was an important opportunity to show Ethiopia's education system from a different perspective. That helped the Ministry of Education and Fine Arts make a number of development works to expand education opportunities across all levels [3]. Many of the tertiary level schools added during this decade were for scientific and technical fields. One of the works of this decade was the revision of secondary school curricula in 1963. The objective of the revision was to make secondary education culturally and locally relevant. As stated in Wagaw [3] one of the objectives stated that:

General secondary school thus defined should be such as to arouse in the young Ethiopian a spirit of inquiry in all field of knowledge, to allow him to appreciate fully his duties as a citizen, to give him a genuine understanding of the national and regional environment, to strengthen his appreciation of his traditional and cultural values; and by making him conscious of the wealth of the world's culture and the continuing progress of science, to give the feeling of being one with the whole of mankind (p. 156).

Very relevant phrases to science and science education such as ‘spirit of inquiry’ and ‘continuing progress of science’ are indicated in this revision statement. But when the recommendations of the various conferences of African states for the development of education about science, science education and technology (Table 3) are examined, the commitment of the Ministry of Education and Fine Arts toward science education was weak. The recommendation of the Addis Ababa Conference about science and science teaching was comparably stronger which would deserve greater attention than the above statement. In fact, it was argued that even though inquiry teaching was part of science education reforms during the Imperial regime, no progress was apparent to promote inquiry [22]. The limitations of trained teachers and science teaching equipment and supplies were so critical to talk about inquiry science teaching if we look into it through the spectacle of the research and practice in science education in many countries of the world. The attempt of providing science lessons through radio and television stations in the mid 1960s [3] shows how the flawed practice of science teaching was deep-rooted. The practice of delivering science lessons through radio and TV stations was still predominant.

**Table 3:** Recommendations of conferences on African science and science education.

Addis Ababa (Ethiopia), 15–25 May, 1961 [10]
• Give greater emphasis to science and its applications in curricular contents
• Promote the development of expertise for teaching scientific and technical subjects
• Make intensive study of science and mathematics to begin at the commencement of the higher stage
• Design science teaching at the higher stage in such a way that it provides systematic knowledge of the material world and appreciation of the nature of science
**Nairobi (Kenya), 16-17 July, 1968** [17]
• Due emphasis be given on training mathematics, science and technology at all levels
• Include language, technology and science sections in teacher training programs
• Take efforts to maintain science and technical graduates in teaching profession
• Provide adequate instruction of science and mathematics for first cycle of second level education. Emphasis to be given to fostering observation and deduction, the study of environment and the application of science to everyday technology.
• Promote the capacity in terms of content knowledge and teaching skills of science teachers at all levels, and other stockholders in science education, scientific enterprise and technical domains
• Calls for Members States to focus on the development of science and technology curriculum
• Recommends introduction of scientific and technical knowledge to the general populace
**Lagos (Nigeria), Jan. 27 – Feb. 4, 1976** [18]
• Correct imbalance in the subject structure and course offerings of postsecondary educational institutions in relation to high-level manpower requirements, especially science and technological skills.
• Assist Member States to strengthen national structures and programs for the promotion and development of science and technological education in Africa
• Explore the feasibility of establishing a team of experts for joint activities in the field of (a) science and technology; and (b) education for development
• Priority be given to programs in international exchange of world literature related to science and technology policies, encouragement of public understanding of science and technology, and studies on the human implications of science and technology
**Harare (Zimbabwe), 28 June-3 July, 1982** [23]
• Introduce integrated science and technology teaching; and create a favorable context, produce appropriate teaching materials, develop relevant instructional methods and train teachers for this purpose in primary and lower secondary levels.
• Establish community centers for the dissemination of scientific and technological knowledge and extension work for non-formal education.
• Apply diversified curricula comprising elementary technical and vocational training in accordance with national development needs, and promote the development of creative activities geared to production in upper secondary education
• Organize interdisciplinary courses of study designed to train supervisory personnel capable of solving the practical problems of the community; develop and improve training for teachers specializing in scientific and technical subjects; and develop appropriate curricula and teaching materials and methods in higher education
• Adopt an interdisciplinary approach to the teaching of science and technology
• Take steps to improve the status of science and technology teachers, bearing in mind the keen competition from the industrial sector in this field
**Dakar (Senegal), 8-11 July, 1991** [24]
• Stressing the importance of science and technology in development, requests for the establishment of the regional committee, namely the committees on higher education, literacy and the teaching of science and technology
• Promote science and technology education and research, in particular research and development in such vital areas as agriculture and the local processing of national resources

#### 3.3.2 Science education in the reign of the Dergue

The various decrees and proclamations related to the education issued by the Dergue regime are indicated in Table 2. It had changed the curriculum of the Imperial education system shortly after it assumed power. Then, it revised its own curricula once more towards the end of the 1980s. The curricula of the Dergue remained functional until it was completely phased out in 2002/03 academic year. However, it didn't issue any officially published education and training policy statement. Though the Dergue had highly politicized the education system and practice of its predecessor, it smartly modified and used the recommendations of the Sector Review and other relevant documents of the Imperial regime [14].

The Dergue had stated its commitment to develop science like other areas of education and training in its famous program of National Democratic Revolution in 1976 [4]. Moreover, after nearly a decade (in 1984), the regime issued a proclamation to strengthen the management and administration of schools, which required the MoE to “ensure that the educational curriculum is prepared on the basis of socialist ideology, and embodies the principle that education given at every level aids to improve the standards of living of the broad masses and *emphasize the development of science and technology*...” (emphasis added) (p. 95) [4]. The establishment of the National Science and Technology Commission in 1975 can also be regarded as important achievement to promote science and technology. But the commission focused on coordinating science and technology research without giving much attention to school science education. When examined from the perspective of the recommendations of the African Education conferences, the efforts of the Dergue to promote science education, where two of them were conducted during its reign, were unsatisfactory (Table 3).

As per the curricula of the Dergue, science was given as General Science from grade 1 to grade 8, Biological and Physical Science for grades 9 and 10, and Biology, Chemistry and Physics for grades 11 and 12 until 1984. All being the same, Physical Sciences were given as Chemistry and Physics starting grade 9 after 1984. The language of instruction for grades 1 through 6 was Amharic (alien to the great majority of linguistically diverse Ethiopian children), whereas for grades 7 through and 12 were taught in English. As a student and a secondary school biology teacher of these curricula, I provided my general observation and impression as follows:

The textbooks of middle school general science, junior and senior high biological sciences, and junior high school physical science of pre-1984 were prepared almost entirely following the various models science inquiry, i.e., confirmation, structured, guided, and independent. However, middle and secondary school teachers of the time rarely teach using any of the models of inquiry. Lessons were taught through chalk and talk. I can only list four instances where I saw science being taught through demonstration and structured inquiry. My first experience was when I was grade 3 (in 1978) where our general science teacher showed us how a flame of a candle is extinguished when it is covered with glass beaker due to lack of oxygen. In the same grade, I remember the same teacher required us to germinate beans and put them in dark, at a window and in light to observe the directions of growth of the seedlings and their colors. Likewise, I remember (in 1982) when my grade 9 biology teacher dissected a frog to show its internal organs as a demo during an annual school day. Lastly, I remember (in 1983) when my grade 10 physical science teacher showed us a demonstration on the diffusion of some iron compound. I had taught grade 9 to 12 biology from 1990 to1994. Admittedly, I had very limited experience of planning and teaching lessons using any of the methods of science inquiry. During that time, I designed and taught only five structured-inquiry activities. These were: the effect of exercise on rate and depth of breathing in relation to gender, the process of osmosis using potato cubes, the internal structure of grasshopper through dissection, demonstrating the functioning of pace-maker in decapitated frog, and dissection of rat to demonstrate the structure of its caecum.

In this respect, some notable studies indicated that even though science curricula were prepared based on inquiry models, they were taught using lecture method. A review by Engida [25] supports my experience. He cited studies that indicated that the teaching of high school biology were highly teacher-centered. Likewise, Bekalo and Welford [14] analyzed chemistry and physics curricula of grades 10 and 11 in relation to promoting active learning and practical activity. They analyzed curricular guides (that indicate policy directions, educational objectives and instructional guidance), textbooks, samples of Ethiopian School Leaving Certificate Examination (ESCLE) and teacher-prepared school exam papers. Their study revealed that curricular documents provided statements of specific objectives. The documents clearly indicated what is expected of the students in terms of cognitive, psychomotor and affective domains at the end of secondary education. Moreover, the objectives emphasized practical approaches of instruction. However, the researchers noted the spread of the activities between cognitive and psychomotor domains and, hence argued that the policy makers and curriculum developers might be unclear about the role of practical work in realizing the three strings of objectives. Bekalo and Welford [14] also noted that even though the curricular guides provided a list of instructional methods that are more appropriate for science teaching, the guides tended to give explanations on how to teach the various topics through teacher demonstration without indicating how to practically engage the students. These researchers suspected that the reason why demonstration was usually recommended as instructional strategy could be because curricular developers were cognizant of the limitations of Ethiopian schools to carry out inquiry or other lab-based lessons. In fact, that was the case. From my experience, there were activities on the detection of CO_2_ through radioactive methods and O_2_ through isotope techniques in grade 11 biology. Hence, the education system of Ethiopia during the Dergue regime was described to be in crisis because of inappropriate instructional methods, which failed to incorporate relevant practical experience [13].

Another important practice of Ethiopia's education system that influenced science education is the Ethiopian School Leaving Certificate Examination (ESLCE). The ESLCE had eroded the value of science and science education and affected the beliefs, practices and attitudes of science teachers and students. If one examined the items of five years' ESLCE, it would be highly likely that a very high proportion of the test items would be repeated at least once. As far as biology and chemistry courses were concerned, students who could solve or study, say 10-years ESLCE papers (ca. 900 multiple choice items each), which were available in school libraries, it was highly likely that majority of them would score A's. Teachers usually opted to solve ESLCE questions, leading students to concentrate on studying and memorizing those questions. Not only the questions were repeated multiple times over the years but also they were low-level memory and comprehension questions. In this case, Bekalo and Welford [14] come up with very important finding. They examined 625 ESLCE test items (225 items of physics and 400 items of chemistry). About 95% of the test items were simple recall items associated with some conceptual understandings. No practice oriented, procedural or application items were included. Exam papers prepared by school teachers revealed similar characteristics to that of the ESLCE items. Bekalo and Welford [22] argued that the problem facing the MoE was how to implement its objectives. In this particular case, the Ministry even failed to correct practices that contradict its objectives.

#### 3.3.3 Science education in the Federal Democratic Republic

As indicated previously, the ETP was put in place in 1994. It was developed based on the argument that the previous system had many problems and failed to provide socially, economically, and culturally relevant and quality education. It is helpful to present some of the justifications for reforming the education system briefly before showing the status of science education system of the Federal Democratic Republic. The major factors were summarized in the report of the International Bureau of Education, Ethiopian National Agency for UNESCO [26], as follows. The previous education systems:

(*a*) Lacked the integration of the curricula with the realities of the learners and the society to guarantee economic and social development;
(*b*) Failed to distinctly indicate the objectives of each level of education in relation to the demands and capacity of the country;
(*c*) Did not present subject area objectives in interrelated and integrated way within the same level or class; which led learners to perceive learning as limited to absorbing facts;
(*d*) Was characterized by mismatches between the socioeconomic realities of the country and the ambitious commitments of the curricula.

The critical issues that were addressed in the ETP were the preparation of explicit educational goals, objective and strategies, the adoption of mother-tongue languages as media of instruction, and the decentralization of educational management and administration. The important change in relation to science education was the structuring of the educational levels. During the Dergue regime, the levels were 6 years of primary, 2 years of middle school and 4 years of secondary. The education system of the Federal Republic divided the educational structure into 8 years of primary and 4 years of secondary. According to the system, science was given as environmental education (for grades 1 through 4), general science (for grades 5 and 6), and as separate fields of biology, chemistry and physics (for grades 7 through 12). Environmental education incorporates both natural and social science contents. The intention of designing an environmental education course was claimed to be for helping learners grasp the applied aspect of science education, i.e., understanding the relationship between science, technology and society [26]. General Science courses in grades 5 and 6 included contents from biological and physical sciences. They were intended to incorporate some aspects of technology. The full-fledged linearity of natural sciences, i.e., biology, chemistry and physics begins in grade 7. In grades 7 and 8, the science courses were planned to incorporate some applications of science and technology. The junior and senior high school science courses incorporated very limited contents of technology as they were regarded as pure science. In any case, the policy supposed that all science courses would enable learners to be capable of problem-solving by understanding their environment. In fact, the policy claimed that the development of natural science curricula was based on contemporary trends of science education, i.e., “to integrate knowledge and application of science, which in turn leads to integration of technology and social issues in science education” (p. 14) [26]. It had also strived to help students improve their problem-solving capacity, hence enabling them to be productive members of their community [15, 16, 21].

For the purpose of drawing a few works and make some reflections, it would be helpful to indicate the commitments of the 1994 education policy and efforts of the MoE to realize its commitments. The policy addressed the issues of science education, i.e., the purposes of science education, the contexts of science curriculum, science teacher education, and the conditions that foster practical in school science [15, 16]. The realization of these objectives could be ensured if appropriate teaching materials were designed, capable science teachers were trained and assigned, and necessary instructional facilities were made available. The strategies of the ETP were supposed to help that these preconditions will be met. Of course, various efforts were undertaken accordingly.

How the levels of education were restructured to offer sound science education programs was indicated above. Following the issuance of the ETP in 1994, the Institute of Curriculum and Development Research of the country and the research and curriculum development departments of the regional states had developed texts and other curricular materials based on the strategies indicated in the policy document [15, 16]. Similarly, teacher education colleges and faculties were established to train teachers as per the policy. The curricula of the teacher education colleges and university faculties were modified to be aligned to the requirements of their trainees. The MoE and state education bureaus had implemented many programs to improve the quality of textbooks and promote the standards of science teacher education. But the teacher education and science text or curricular preparations were marred with problems.

The crux of the problem of science education began in the in-service and pre-service teacher education institutes. Researchers argued that the training of pre-service and in-service science teachers in the teacher education colleges of the country was characterized by too much factual information, lower order cognitive skills, little inquiry activities, and mismatches between theoretical and practical knowledge [25]. Bekalo and Welford [22] asserted that science educators in the Ethiopian teacher education colleges were not educated in such a way that they could help their pre-service and in-service trainees develop science skills and processes. Therefore, a mismatch had grown between what was often planned in the policy and the instructional tradition in the schools and colleges. The authors further proclaimed that teachers (and teacher educators) continued to use teacher-centered teaching strategies against the requirements of achieving the goals of the policy [22]. Even science educators had difficulties in understanding the concept of problem-solving approach.

The present authors had been teaching at one of the teacher education colleges of the country. The course catalogs for science trainees (i.e., biology, chemistry and physics) included a single, two credit hour course in subject methodology. Most of the instructors believed that the science method course is added simply for credit requirements. The major focus of the modifications of the teacher education curricula was for providing strong science courses rather than offering sufficient training in science education. In this respect, one participating teacher in the study of Bekalo and Welford [14] stated that:

Most of us here [in the teacher education college] are pure Physics, Chemistry, and Biology graduates. ...We took a few educational methodology courses at university, but that is not enough and we can't say we are educators. Because of our background of our college courses (on which we teach) are highly influenced by the university course we brought with us. You can compare the contents yourself. On the contrary, we do not relate the content to the school curriculum and I have never even seen any school curriculum materials (and I have been) working here for years. I think there is a clash between what we teach and the objectives of the college to train teachers for schools (p. 204).

The quality of the preparation and the contents of science textbooks are targets of criticism. Even though student-centered instructional strategies were highly recommended, and usually praised, the selection of contents and the preparation of the textbooks did not support active hands-on and minds-on involvement of students. A study of grade 7 chemistry textbook by Engida [27] revealed that the attempts to provide relevant curriculum were unsuccessful. The main problems identified in the study of Engida [27] were: limitations in incorporating appropriate contents and instructional strategies and contextualizing the curriculum, giving high emphasis on theoretical declarative concepts, failure in considering the cognitive development of the students, and failure to include contents that were locally relevant and apparent. It might have been possible to train teachers who were capable of using a repertoire of science teaching methods. But failing to provide textbooks and other curricular materials that were suitable for learner-centered instructional practice rendered science teachers helpless. The nature of students' textbooks and other curricular materials influence the teaching behavior of teachers. In this regard, an observation of brief case study by this author is provided below.

Case Study: Science Texts of Tigrai State.

Summary of simple descriptive analysis of upper primary science textbooks of Tigrai State is given in Table 4 as representative of upper primary science textbooks of all other regional states of Ethiopia. Since the curricular frameworks and guidelines were developed by the Ministry of Education of the Federal government, the contents of the curricula of all other national regional states were similar. In fact, the curricular contents of upper primary and secondary school science of all the regional states are exactly the same except the languages they are written in. All are copied from the same English version. Therefore, observations about the quality of upper primary science textbooks of Tigrai are applicable to the science textbooks in all regional state without exception.

**Table 4:** Inquiry activities in upper primary science textbooks of Tigrai [28, 29, 30, 31, 32, 33, 34, 35].

Subject	Grade	Text Size (VG Main 12 Points)				Types of Activities ^(a)^				
		Pages	Units	Paper Size (In.)	Graphics	CI	SI	GI	UI	Total
General Science	5	131	7	8.27 × 11.69	150	15	11	–	–	26^(b)^
General Science	6	177	9	8.27 × 11.69	156	15	13	–	–	28^(c)^
Biology	7	209	6	6.50 × 8.50	108	20	–	–	–	20
Biology	8	298	6	6.50 × 8.50	78	15	–	–	–	15^(d)^
Chemistry	7	203	5	6.50 × 8.50	62	20	–	–	–	20
Chemistry	8	352	5	6.50 × 8.50	50	33	–	–	–	33
Physics	7	340	10	6.50 × 8.50	109	20	3	–	–	23
Physics	8	340	6	6.50 × 8.50	181	19	–	–	–	19
Total	56		54		894	157	27	–	–	184

^(a)^ = CI: confirmation inquiry; SI: structured inquiry; GI: guided inquiry; UI: unguided or independent inquiry.

^(b)^ = 18 of the inquiry activities were from Unit 7. Two of the seven units did not include any inquiry activity.

^(c)^ = Four of the nine units did not have any inquiry activity.

^(d)^ = Three of the six units did not have any inquiry activity.

It is apparent that the volumes of the science curricula (i.e. textbooks) were quite overwhelming. The summary can assist readers to give reasonable estimates of the amount of contents of the curricula. The number and types of inquiry activities in each of the textbooks are also provided. This simple investigation revealed that, in theory, students would have the opportunity to be engaged in less than 190 science inquiry activities over four years of upper primary schooling. Over 85% of these activities were *ill-designed* simple confirmation activities in which students *would* verify known information or principles.

**Figure 1:**
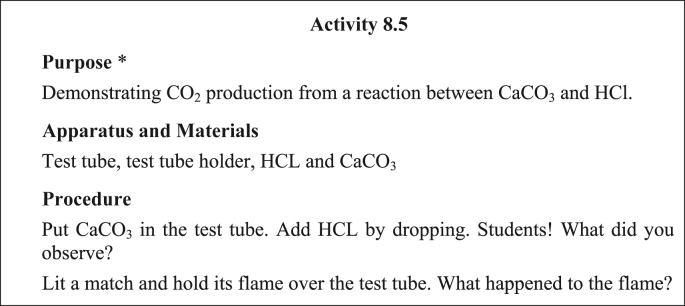
Activity in Grade 6 General Science Textbook of Tigrai State, Ethiopia (Tigrai State Education Bureau (TBE), 1998; p. 142) [31] ∗ The purpose of the activity was not clearly indicated as it appeared here. It is inferred from the procedure. Neither was the format.

Most of the inquiry activities were designed for the purpose of factual demonstration such as microscopic observation of amoeba and testing acid using litmus paper or for teaching techniques such as separation of salt and sand or setting up an electric circuit. But the expected outcomes of nearly all of the activities were often indicated in the textbooks. Unfortunately, many of them were difficult to execute due to the absence of inputs and lack of technical skills of the teachers. For example, Activity 8.5 in grade 6 General Science was intended for demonstrating CO_2_ production from a reaction between CaCO_3_ and HCl (p. 142) [31] (Figure 1). But no elementary school existed in the State with facilities to support this kind of activities.

Several limitations can be listed about this example and many other similar activities. Nevertheless, the intention of providing this example is to indicate that the apparatus and chemicals listed in the activity and many other inquiry activities provided in the textbooks *never existed* in primary schools of Tigrai State. This shows us that there were serious problems in preparing instructional materials for teaching the processes and products of science. Other problems were indicated in Engida's study of grade 7 chemistry textbook for Addis Ababa city [27]. The mismatches between what were indicated in curricular objectives and what were at stake for the learners and teachers were very apparent. Hence, as far as upper primary school science education system was concerned, what had been promised in the policy was never translated into classroom practices. In fact, the way the textbooks were prepared was contrary to the critical commitment of the policy – a paradigm shift from teacher-centeredness to student-centeredness. Crafting a good policy was one step forward. Unfortunately, the attempts to prepare textbooks that support the objectives of the policy were unsuccessful – two steps backward.

One of the arguments of reforming the education system of the Dergue regime was because *it lacked the integration of the curricula with the realities of the learner and the society* to guarantee economic and social development (emphasis added). From the perspective of the learners, this argument implied that, in the Dergue system, there existed mismatches between what were indicated in the textbooks and the realities of the learner. What did it mean by ‘realities of the learner’? Science curriculum developers (or science textbook writers) should consider at least two sets of realities of learners – spatial-temporal realities and cognitive-developmental realities. Spatial-temporal relevance and cognitive-developmental appropriateness of the contents of upper primary science texts of Tigrai State could have been a play yard of hot contention. For the purpose this article, few examples that showed the lack of integration of the content with the realities of the learners are provided.

Unit 1 of grade 6 General Science focused on problems of the environment [31]. A topic of this unit dealt with air pollution. It talked about how SO_2_, NO_2_, CO and CO_2_ are produced and pollute the atmosphere and the environment in general. However, the problem of air pollution was neither spatially nor temporally relevant for a student from Tigrai in the 1990s. It might not even relevant for a student of Addis Ababa city. Unit 5 of the same textbook dealt with the solar system and covered topics on various constellations of stars – remotely relevant to the realities of the students. A third and final example is interesting. It was a unit on human reproduction (Unit 6). The unit covered topic on human primary and secondary sexual characteristics, human sexual organs, menstruation and menstrual cycle, pregnancy and birth, health complications and problems of female reproduction, abortion, genital mutilation, sexually transmitted diseases, prevention of sexually transmitted diseases and birth control (contraceptive) methods. According to the Ethiopian education system, the age of a 6 grader is 12–13 years. It is apparent that the topics of this unit were too early for 6 graders, hence temporally inappropriate. These showed that the textbook writers ended up designing textbooks that did not consider the realities of the learners both in terms of place (space) (air pollution was not locally relevant) and time (human reproduction is too early for 12 to 13-years-olds). These contents were difficult to contextualize for Ethiopian children of that level.

The second set of problems was the mismatches between the contents and the cognitive development of the learners. Unit 4 of grade 6 General Science dealt with refraction. Some of the contents that were abstract for 12 to 13-year-olds were focal point, real image and how concave and convex lens change the angles of light rays. Whether 12 to 13-years-olds would learn these topics without difficulty or not is debatable. In fact, developmentally and cognitively inappropriate contents were very common. Without going too far to give details, as indicated in Table 4, the contents included in each textbook were too bulky for primary school students. Hence, in this case too, the commitment of the policy and the anticipations in the curricular objectives were difficult to realize.

As observed in this mini case study and recommended by other researchers [8], improving the quality of textbooks was one of the areas special attention. The report by the Ethiopian National Agency for UNESCO of International Bureau of Education, in March 2001, noted the prevalence of the problems thus far discussed [26]. According to that report, the problems pertinent to science education, in addition to low level of literacy and higher gender gap in favor of males were:

(*a*) Shortage and sometimes lack of appropriate kits and laboratory facilities in primary and secondary schools for teaching natural science.
(*b*) Difficulty of reorienting the educational system towards problem-solving and critical-thinking approach to help for economic development.
(*c*) Difficulty of ensuring a paradigm shift from teacher-centered teaching tradition to student-centered instructional methods because of economic and social problems.

The MoE had stressed on the above problems during the preparation of a Program Action Plan for Education Sector Development Program II (2002/3 to 2004/5) [21]. According to that document, there appeared a serious problem in effectively employing the strategies of the policy to realize the general and specific educational objectives. Based on the preceding observation and the reports given by the Ethiopian National Agency for UNESCO, the International Bureau of Education [26] and the MoE [21] as well as the findings of Bekalo and Welford [14] and Engida [27], the status of the link between each of the educational strategies and the primary and secondary school science learners could be described as *weak*. The ETP had prescribed 70 strategic directions, many of which aimed at promoting science education [15, 16]. But in the Tigrai Regional National State, only eight (11.4%) were fully implemented, 36 (51.4%) were partly implemented, and 26 (37.2%) were not attempted at all. Seven of the eight (87.5%) implemented strategic directions were simple changes such as dividing the educational level into eight years of primary and four years of secondary. With overwhelming student populations in the larger regional states such as Oromia, Amhara, and Southern Nations, Nationalities and Peoples and with low socioeconomic development in the smaller and peripheral regional states such as Somali, Afar, Gambella, and Benishangul-Gumuz states, the implementation of the 1994 ETP would not be different from the case in Tigrai.

## 4 Concluding remarks and recommendations

Many of the problems and challenges that prevented the country from building relevant and quality science education were the resultant of the low socioeconomic status of the country. Thus, they were expected to be solved step by step. Nonetheless, one subject must have been addressed at any rate – the curricular materials. The *core* of the educational policy of the country was the development relevant and quality science curricula, though that might not have guaranteed their appropriate implementation. There were nine overall strategies to realize the objectives of the 1994 education and training policy. For instance, the principal purpose of the strategies was to provide learners with curricula that help them improve their problem-solving capacities and make them more productive members of their communities [21]. Hence, eight of the strategies of the education and training policy were supposed to support and enhance the processes of development and implementation of relevant and quality curricula that can produce literate and productive citizens − i.e. relevant and quality education that reflects and responds to individual and social needs, expectations, and aspirations of the citizens. As far as science education was concerned, as presented in many research and government reform documents, the Federal Republic and the states were only partly successful in developing and implementing relevant and quality curricula until the dawn of the 21 century.

This article has attempted to show where the critical challenges in building relevant and quality science education lie. Unfortunately, nearly a lifetime of one generation in the 21^st^ century just gone, science teachers and educators, school administrators, policy-makers, students, and the general public at large are still calling for relevant and quality education, in general, and relevant and quality science education, in particular as detailed in the status report documented to prepare the Education Development Roadmap (2018–2030) [1]. Therefore, with the same challenges still lingering, future policy and legal frameworks as well as education development initiatives that emanate from such frameworks need to critically look into the historical endeavors, achievements, and challenges to acquire a good deal of knowledge in the subject. Such an approach assists policy-makers and development agencies in formulating science-based, research-informed policy directives and development initiatives.

## Declarations

### Author contribution statement

Desta Berhe Sbhatu: Development and wrote this article.

### Funding statement

This research did not receive any specific grant from funding agencies in the public, commercial, or not-for-profit sectors.

### Data availability statement

Data included in article/supplementary material/referenced in article.

### Declaration of interests statement

The authors declare no conflict of interest.

### Additional information

No additional information is available for this paper.
